# Editorial: Post-transcriptional and Post-translational Regulation of Cancer Metabolism

**DOI:** 10.3389/fcell.2021.779157

**Published:** 2021-11-17

**Authors:** Bin Yuan, Qinong Ye

**Affiliations:** ^1^Department of Pharmacology, School of Basic Medical Sciences, Anhui Medical University, Hefei, China; ^2^Department of Medical Molecular Biology, Beijing Institute of Biotechnology, Beijing, China

**Keywords:** cancer metabolism, regulation, microRNA, transcription, metabolic reprograming

The metabolic reprogramming of cancer cells is required for cancer cell growth, migration, and invasion. The common phenomenon of this altered metabolism, also termed the Warburg effect or aerobic glycolysis, is elevated glucose uptake and lactate production, as well as utilization of amino acids and lipids (Koppenol et al., 2011; Liberti and Locasale, 2016; Li et al., 2018). Metabolic reprogramming is a well-recognized hallmark of cancer biology (Hanahan and Weinberg, 2011). The metabolic regulation of cancer cells is controlled by intrinsic genetic mutations and/or external responses to the tumor microenvironment (TME) (Cairns et al., 2011). Expression of metabolism-related genes can be regulated at multiple levels, including transcriptional, post-transcriptional, and post-translational levels. However, knowledge on the regulation of cancer metabolism at the post-transcriptional and post-translational levels is limited. This Research Topic “Post-Transcriptional and Post-Translational Regulation of Cancer Metabolism” provides articles on the fast-growing field of cancer metabolism, where 10 research articles and one review are presented, although most articles focus on transcriptional regulation of cancer metabolism.

Transcription factors, such as the oncoproteins c-Myc (Kim et al., 2004) and HIF-1α (Denko, 2008), have been shown to regulate cancer metabolism by directly binding promoters of metabolism-related genes and controlling the expression of metabolism-related genes. In this topic, Yang et al. (a) shows that c-MYC increases cholesterol biosynthesis and enhances cancer cell proliferation through transcriptional upregulation of SQLE, a rate-limiting enzyme in the cholesterol synthesis pathway. Similarly, Che et al. discovered that the MYC family members c-MYC/MYCN are involved in Keratin 6A-mediated upregulation of glucose-6-phosphate dehydrogenase (G6PD), the rate-limiting enzyme of the pentose phosphate pathway (PPP), leading to enhanced PPP flux in lung cancer cells. The authors of these two articles each present novel mechanistic insights into the c-Myc regulation of cancer cell metabolism, and provide potential targets or approaches for selectively targeting c-Myc-driven metabolic reprogramming in cancer cells. The study by Yang et al. (b) discusses how the F-box protein JFK is a novel transcriptional target of HIF-1α and mediates HIF-1α-induced glycolysis in breast cancer. JFK promotes mammary tumor initiation and progression in the MMTV-PyMT murine spontaneous mammary tumor model. Reactive oxygen species (ROS) are a crucial determinant of cancer metabolism phenotype, and glutathione (GSH) biosynthesis is required for cellular redox homeostasis, which provides energy support for cancer cell growth. As illustrated in the study by Zhao et al., the transcription factors CREB1 and ATF1 negatively regulate GSH biosynthesis by suppressing the transcription and expression of glutamate-cysteine ligase modifier subunit (GCLM) and glutathione synthase (GSS), and thereby dampen the cellular ability to scavenge ROS, resulting in sensitizing cancer cells to oxidative stress. Dong et al. identified recombination activating genes 1 (Rag1) and Rag2 as novel Notch1 transcriptional targets in acute T-cell lymphoblastic leukemia (T-ALL) cells. Dimeric Notch1 transcriptional complexes stimulate Rag1 and Rag2 expression via a novel cis-element harboring a sequence-paired site. Although Notch1 was shown to be involved in cancer glycolysis, it remains unclear whether Rag1 and Rag2 regulate glycolysis in T-ALL cells.

Metabolic reprogramming not only results from the dysregulated expression of diverse genes but also the altered expression of non-coding RNAs (ncRNAs), including microRNAs (miRNAs). miRNAs are involved in several metabolic and tumorigenic pathways through their post-transcriptional regulatory mechanisms. Ye et al. showed that miR-16-1-3p inhibits expression of PGK1 (Phosphoglycerate kinase 1), the first adenosine triphosphate (ATP)-generating glycolytic enzyme in the aerobic glycolysis pathway, by directly targeting PGK1 3'-untranslated region, resulting in decreased glucose uptake, lactate, and ATP production, and extracellular acidification rate, and increased oxygen consumption rate. Aerobic glycolysis modulated by the miR-16-1-3p/PGK1 axis is important for controlling breast cancer cell proliferation, migration, invasion, and metastasis.

Post-translational modifications of key metabolic enzymes, such as phosphorylation, acetylation, ubiquitination, and sumoylation, are also implicated in cancer metabolic reprogramming by changing the activity and/or stability of the enzymes. Li et al. analyzed the expression of 15 SUMOylation regulators in glioblastoma and found that single-nucleotide variant mutations exist in 10 SUMOylation regulators (SENP7, SENP3, SENP5, PIAS3, RANBP2, USPL1, SENP1, PIAS2, SENP2, and PIAS1). The SUMOylation regulator-related molecules (ATF7IP, CCNB1IP1, and LBH) had a strong predictive ability for the overall survival of patients with glioblastoma. However, whether these SUMOylation regulators and SUMOylation of their substrates regulate cancer metabolism remains to be investigated in glioblastoma.

In addition to the regulation of cancer metabolism by altered metabolic enzymes and their upstream regulators, alterations in downstream metabolites are also important for metabolic reprogramming in cancer. Bai et al. analyzed serum metabolites by targeted metabolomics and screened urea, a by-product of ammonia metabolism, as a potential biomarker for HCC. Sepsis may occur in some patients with cancer. Li et al. performed a comprehensive analysis with plasma metabolic profiling of pediatric sepsis, especially identifying a panel of metabolites alteration associated with pediatric sepsis. The authors also discussed energy and carbohydrate metabolism may contribute to sepsis progression. However, these two articles lack molecular mechanisms by which altered metabolites are regulated.

Importantly, the metabolic phenotype of a given tumor is largely influenced not only by the tumor cells themselves but also by the tumor microenvironment. Zhang et al. screened prostaglandin-endoperoxide synthase 2 (PTGS2) as a potential therapeutic target from TCGA-UVM (uveal melanoma) dataset. Decreased tumor cell proliferation and increased apoptosis was observed with the treatment of PTGS2 inhibitor, celecoxib. In an extensive review, Wang et al. focus on the inhibitory effects of cancer-associated fibroblasts (CAFs) and on cancer development, including heterogenous CAFs metabolism.

The current collection mainly focuses on the genetic changes that alter cancer cell metabolism and lack research articles on the abnormal TME, which play an important role in determining the metabolic reprogramming of cancer cells. On the other hand, the current collection does not cover all the topics proposed in the open call for submissions and still lacks a deeper understanding of post-transcriptional and post-translational regulation of cancer metabolism. For instance, it remains unclear how RNA binding proteins and ncRNAs regulate mRNA stability of metabolic enzymes, and how metabolic enzymes, at least some metabolic enzymes, are post-translationally modified, thus regulating cancer metabolism. However, the insights gathered in the present Research Topic nevertheless strengthen understanding of the regulation of cancer metabolism as an important area in cancer biology research.

## Author Contributions

All authors listed have made a substantial, direct and intellectual contribution to the work, and approved it for publication.

## Conflict of Interest

The authors declare that the research was conducted in the absence of any commercial or financial relationships that could be construed as a potential conflict of interest.

## Publisher's Note

All claims expressed in this article are solely those of the authors and do not necessarily represent those of their affiliated organizations, or those of the publisher, the editors and the reviewers. Any product that may be evaluated in this article, or claim that may be made by its manufacturer, is not guaranteed or endorsed by the publisher.
